# Reduction of CPR artifacts in the ventricular fibrillation ECG by coherent line removal

**DOI:** 10.1186/1475-925X-9-2

**Published:** 2010-01-06

**Authors:** Anton Amann, Andreas Klotz, Thomas Niederklapfer, Alexander Kupferthaler, Tobias Werther, Marcus Granegger, Wolfgang Lederer, Michael Baubin, Werner Lingnau

**Affiliations:** 1 University Clinic of Anesthesia, Innsbruck Medical University, Anichstr 35, A-6020 Innsbruck, Austria; 2 Faculty of Mathematics, University of Vienna, Nordbergstr 15, A-1090 Vienna, Austria

## Abstract

**Background:**

Interruption of cardiopulmonary resuscitation (CPR) impairs the perfusion of the fibrillating heart, worsening the chance for successful defibrillation. Therefore ECG-analysis *during ongoing chest compression *could provide a considerable progress in comparison with standard analysis techniques working only during "hands-off" intervals.

**Methods:**

For the reduction of CPR-related artifacts in ventricular fibrillation ECG we use a localized version of the *coherent line removal *algorithm developed by Sintes and Schutz. This method can be used for removal of periodic signals with sufficiently coupled harmonics, and can be adapted to specific situations by optimal choice of its parameters (e.g., the number of harmonics considered for analysis and reconstruction). Our testing was done with 14 different human ventricular fibrillation (VF) ECGs, whose fibrillation band lies in a frequency range of [1 Hz, 5 Hz]. The VF-ECGs were mixed with 12 different ECG-CPR-artifacts recorded in an animal experiment during asystole. The length of each of the ECG-data was chosen to be 20 sec, and testing was done for all 168 = 14 × 12 pairs of data. VF-to-CPR ratio was chosen as -20 dB, -15 dB, -10 dB, -5 dB, 0 dB, 5 dB and 10 dB. Here -20 dB corresponds to the highest level of CPR-artifacts.

**Results:**

For non-optimized *coherent line removal *based on signals with a VF-to-CPR ratio of -20 dB, -15 dB, -10 dB, -5 dB and 0 dB, the signal-to-noise gains (SNR-gains) were 9.3 ± 2.4 dB, 9.4 ± 2.4 dB, 9.5 ± 2.5 dB, 9.3 ± 2.5 dB and 8.0 ± 2.7 (mean ± std, *n *= 168), respectively. Characteristically, an original VF-to-CPR ratio of -10 dB, corresponds to a variance ratio *var*(VF):*var*(CPR) = 1:10. An improvement by 9.5 dB results in a restored VF-to-CPR ratio of -0.5 dB, corresponding to a variance ratio *var*(VF):*var*(CPR) = 1:1.1, the variance of the CPR in the signal being reduced by a factor of 8.9.

**Discussion:**

The *localized coherent line removal *algorithm uses the information of a single ECG channel. In contrast to multi-channel algorithms, no additional information such as thorax impedance, blood pressure, or pressure exerted on the sternum during CPR is required. Predictors of defibrillation success such as mean and median frequency of VF-ECGs containing CPR-artifacts are prone to being governed by the harmonics of the artifacts. Reduction of CPR-artifacts is therefore necessary for determining reliable values for estimators of defibrillation success.

**Conclusions:**

The *localized coherent line removal *algorithm reduces CPR-artifacts in VF-ECG, but does not eliminate them. Our SNR-improvements are in the same range as offered by multichannel methods of Rheinberger et al., Husoy et al. and Aase et al. The latter two authors dealt with different ventricular rhythms (VF and VT), whereas here we dealt with VF, only. Additional developments are necessary before the algorithm can be tested in real CPR situations.

## Background

Frequent interruptions of chest compressions (CC) as part of cardiopulmonary resuscitation (CPR) during ventricular fibrillation (VF) and pulseless ventricular tachycardia (VT) impair myocardial perfusion and worsen the chance for successful defibrillation with stable return of spontaneous circulation [1,2]. Eilevstjonn et al. reported that "no-flow times" (NFT) comprise about 50% of time during resuscitation [3], and gave suggestions on how to reduce NFT. On the other hand, analysis of the ECG for fibrillation detection necessarily requires interruption of CC, at least with the ECG-analysis algorithms currently implemented in defibrillators available on the market.

Therefore ECG-analysis *during ongoing chest compression *can provide a considerable progress in comparison with standard analysis techniques working only during "hands-off" intervals [4]. These intervals have recently been found to be unexpectedly long and harmful [1,5,6]. In addition to avoiding "hands-off" times, new analysis techniques could become the pre-requisite for prediction of defibrillation success probability [7-18]. These analysis techniques would allow to avoid unpromising and therefore ultimately damaging defibrillator shocks.

Aase, Husoy, Eilevstjonn et al. [19-22] have developed adaptive filtering approaches to real-time separation of VF/VT and CPR for a multi-channel-context. Berger et al. suggested an adaptive noise cancellation technique [23] and recently Kalman filtering techniques were used [4,24]. We note also the contributions by Aramendi et al. [25] and Irusta et al. [26]. In the present work, we concentrate again on time-frequency methods [27,28] and on the situation where only one ECG channel is available, without any additional information concerning blood pressure or concerning the pressure on the sternum applied during resuscitation. This is particularly adapted to the current use of automated external defibrillators (AEDs), where - so far - no such additional information is available.

In this work we present a method based on a time-frequency analysis. The method makes use of a *windowed Fourier transform *that captures characteristic features of VF signals and CPR artifacts. A commonly used criterion to assess an algorithm is the improvement of the signal-to-noise ratio (SNR). The SNR is expressed as the variance of the "proper" VF-ECG without CPR-artifacts (signal) divided by the variance of the CPR-artifacts (noise) in the ECG. *Coherent line removal *can be used for reduction of CPR-related artifacts in VF signals, because the fibrillation ECG does not contain a line spectrum (at some particular frequency) which would unintentionally be removed by the algorithm, but many different frequencies which are continuously distributed in a "fibrillation (frequency) band".

Mere improvement of signal-to-noise ratio is not a guarantee for a better estimate of the "proper" VF-ECG. It is of additional importance, that typical ECG-based parameters like the median frequency (of the ECG) show similar results in estimate_VF_(*t*) as compared to ECG_VF_(*t*). Furthermore, it is important that an artifact-free ECG-signal (containing VF only) shows approximately the same median frequency before and after application of the CPR-filtering algorithm. Median frequency is considered to be an interesting parameter for prediction of defibrillation success [8,10,29], even though not unequivocally [30,31].

In the present work, we illustrate by examples how the proposed method affects the median frequency and present numerical results for the SNR-improvement.

## Methods

### (A) Data

We used one exemplary dataset from a VF-experiment in a pig model for illustration of the effect of our CPR-reduction algorithm on the power spectrum. Data in this animal experiment were recorded with 12 bit and 1000 Hz sampling frequency. For the present purpose, we used a downsampled version of the ECG recorded with 200 Hz sampling frequency.

The actual testing of our CPR-reduction algorithm was done with 14 different human ventricular fibrillation (VF) ECGs, which were mutually mixed with 12 different ECG-CPR-artifacts recorded in an animal experiment during asystole with an applied CPR-frequency between 80/min and 120/min. The length of each of the ECG-data was chosen to be 20 sec, and testing was done for all 168 = 14 × 12 pairs of data.

The 14 different human ECGs have been collected using a Welch Allyn PIC 50 defibrillator, recorded with 12 bit and 375 Hz sampling frequency during real out-of-hospital CPR situations. For the present purpose we chose human ECGs with frequencies lying (roughly) in the range [1 Hz, 5 Hz].

The pig experiments were conducted according to Utstein-style guidelines [32] and approved by the Federal Austrian Animal Experiment Committee. The recording of the human data was approved by the local Ethics Committee of Innsbruck Medical University.

### (B) Data processing and quality assessment for CPR-reduction algorithms

Data were processed using MATLAB (The Mathworks, Natick (MA), version R2007b). For computation of mean frequency, median frequency and dominant frequency the upper cut-off frequency was 30 Hz. The lower cut-off frequency was 4.33 Hz (for ECG-data including CPR-artifacts) and 2.2 Hz (for ECG-data purged from CPR-artifacts).

For spectrograms shown in Figures the frequency range has been restricted to [0 Hz, 15 Hz] to improve visibility (the spectrogram above 15 Hz does not contain much interesting information). The "typical frequencies" corresponding to ventricular fibrillation ECG are called the "fibrillation band". This "fibrillation band" changes in the course of time. For human VF-ECGs, the fibrillation band typically is within the frequency window [1 Hz, 6 Hz].

In an ECG-signal

composed of a CPR-artifact-free VF-ECG and an ECG containing only CPR-related artifacts, the SNR is defined as

where var(ECG_VF_) is the variance of the proper VF-ECG and var(ECG_CPR_) is the variance of the CPR-related artifacts in the ECG. The acronym *SNR *refers to "signal-to-noise ratio", where the signal is ECG_VF _and the noise is ECG_CPR_. Usually it is expressed in decibel units (dB), i.e., as 10 times the logarithm (with basis 10) of this SNR. For very strong artifacts, the signal-to-noise ratio is around -20 dB, whereas 0 dB corresponds to rather small CPR-related artifacts.

An *algorithm for extraction of CPR-artifacts *in an ECG gives rise to a decomposition

The quality of such an algorithm can be assessed by looking at the variance of error

or by considering the restored signal-to-noise ratio

In particular, we use the SNR-gain, i.e., the difference (rSNR - SNR).

### (C) Coherent line removal

**Figure 1:**
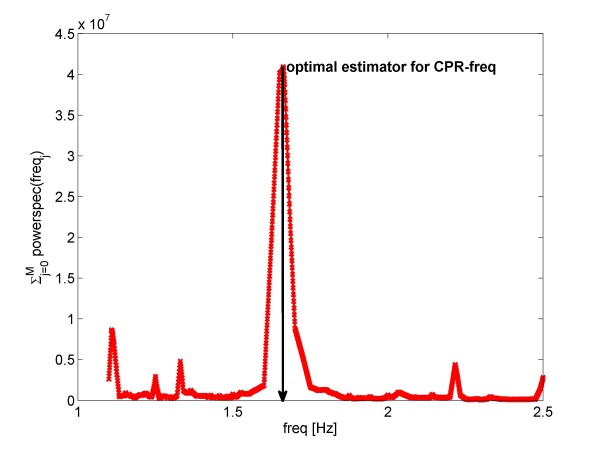
**This figure illustrates the way in which a first estimator for the coherent line removal algorithm is chosen**. The function  is displayed for an ECG-signal *s *= *s*(*t*) containing CPR-artifacts. The optimal estimator for the CPR-frequency is taken as the frequency which maximizes this function. Here *f *is an arbitrary frequency and (*kf*), *k *= 2,3, ..., *M*, are its harmonics. The function *ŝ *= *ŝ*(*f*) is the Fourier transform of the ECG-signal *s *= *s*(*t*) and |*ŝ*(*f*)|^2 ^is the powerspectrum. In the present example the optimal estimator *f*_0 _for the CPR-frequency is *f*_0 _= 1.66 Hz.

For the reduction of CPR-related artifacts we used the *coherent line removal *algorithm developed by Sintes and Schutz [28,33,34]. This method can be used for removal of periodic signals with sufficiently strong harmonics, and is presented in more detail in the Appendix. Before applying coherent line removal to a signal *s *= *s*(*t*), the approximate CPR-frequency is estimated from the sum of power at (each) frequency *f *and its harmonics, taking the frequency *f*_0 _at which the following function maximizes:

The function *ŝ *= *ŝ*(*f*) is the Fourier transform of the ECG-signal *s *= *s*(*t*). Furthermore *f *is some chosen frequency, and (*k f*), *k *= 2,3, ..., M, are its harmonics. An example, plotting this function for an ECG containing CPR-artifacts is shown in Fig 1.

Coherent line removal has a few parameters which can be adapted to the signal in question:

• the time window,

• the number *har *of harmonics (default = 4),

• the frequency width *delta *for line removal (default = 4),

• and the number of harmonics *NumHar *considered for reconstruction (default = 10),

We choose a time window of 2048 data points, corresponding to 10.24 sec (at a sampling frequency of 200 Hz), and determined the optimal choice of *har*, *delta *and *NumHar *in a grid search, varying *har *and *NumHar *from 2 to 10, and *delta *from 2 to 5 (or took default values). The "grid search" simply computes the variance of the error var(ECG_CPR _- estimate_CPR_) and looks for the smallest error (among all different sets of parameters) for our 168 = 14 × 12 datasets. Minimization of var(ECG_CPR _- estimate_CPR_) is equivalent to maximum SNR improvement.

## Results

**Figure 2:**
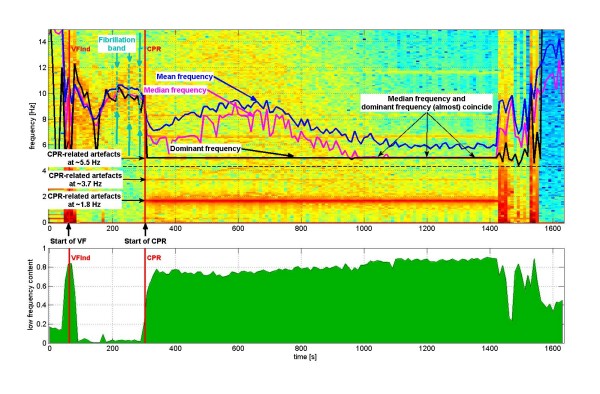
**Windowed Fourier transform of ECG for one particular chosen VF-experiment in a pig model**. The power (in units of decibel [dB]) is shown in a color coded spectrogram (upper panel), with 'red' representing high power, 'yellow' intermediate power and 'blue' low power. Time [sec] is given on the x-axis, frequency [Hz] on the y-axis. Fibrillation starts at ~80 sec, and cardiopulmonary resuscitation (CPR) after ~310 sec. CPR is performed with approximately ~110/min = ~1.8 Hz. The respective artifacts at ~1.8 Hz are clearly visible in the spectrogram, together with the harmonics ~3.7 Hz, ~5.5 Hz etc. The "fibrillation band" corresponding to VF-ECG during the first phase of the experiment shows the typical "S-form", starting at ~10 Hz, decreasing to ~8 Hz, and increasing again during the first 240 sec of VF. Afterwards, the "fibrillation band" *would *continuously decrease *if *no CPR were performed. CPR increases the frequency range of the "fibrillation band". Three different parameters are computed for the VF-ECG with respect to the frequency window [4.33 Hz, 30 Hz]: mean frequency (blue), median frequency (magenta), and dominant frequency (black). The lower frequency of the frequency window (= 4.33 Hz) is shown as a straight dashed black line. Before start of CPR, the mean, median and dominant frequency follow the "fibrillation band". *After *start of CPR, the dominant frequency remains at the second harmonic of CPR-frequency at ~5.5 Hz. In addition (*after *start of CPR), the mean and median frequency do not at all follow the "fibrillation band" but are rather influenced by the harmonics of the CPR-artifacts. The lower panel of the figure shows the relative power in the frequency window [0.33 Hz, 2 Hz] as compared to the frequency window [0.33, 30 Hz], denoted as the "low frequency content" of the signal.

Fig 2 shows the windowed Fourier transform of an ECG for a VF-experiment in a pig model. Fibrillation in this example starts at ~80 sec, and cardiopulmonary resuscitation (CPR) after ~310 sec. CPR is performed with approximately ~110/min = ~1.8 Hz. The respective CPR-related artifacts at ~1.8 Hz are clearly visible in the spectrogram, together with the harmonics at ~3.7 Hz, ~5.5 Hz etc. The mean and median frequency computed for the frequency window [4.33 Hz, 30 Hz] are good parameters for the "fibrillation band" as long as no CPR is performed [8,10]. As soon as CPR starts, the mean and median frequency do not reflect the course of the "fibrillation band" any more. The "fibrillation band" is *increasing *after start of CPR, whereas the mean and median frequency are *decreasing *after start of CPR. Starting from ~1000 sec in this example, the median frequency is completely dominated by the second harmonic of the CPR-artifacts at ~5.5 Hz.

**Figure 3:**
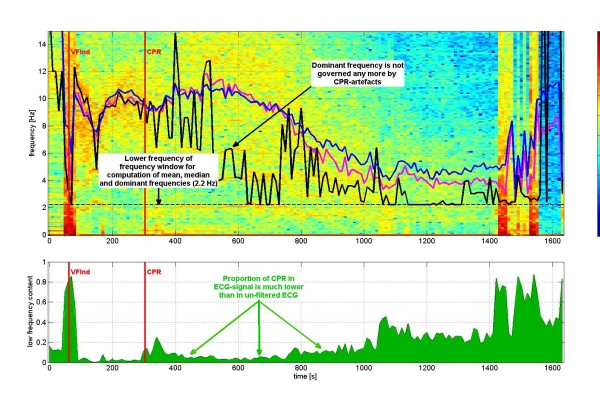
**Windowed Fourier transform with the same basic ECG as used in Fig 2, but now for the VF-ECG purged from CPR-artifacts by *coherent line removal *and with a frequency window of [2 Hz, 30 Hz] for determination of mean, median, dominant and 95%-edge frequency**. The CPR-related artifacts are barely visible in the spectrogram. The mean and median frequency follow the "fibrillation band" rather well, apart from the last stage of the experiment (> 1100 sec), where no "fibrillation band" is visible any more (probably due to asystole). The lower panel indicates that the relative power in the frequency window [0.33 Hz, 2.2 Hz] as compared to the frequency window [0.33, 30 Hz] is much smaller now than in Fig 2.

**Figure 4:**
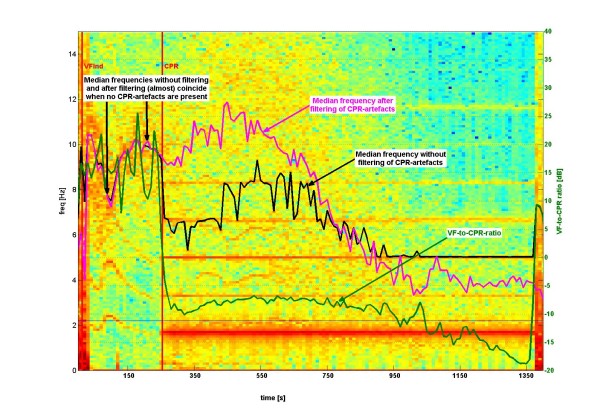
**Windowed Fourier transform with the same basic ECG-data as in Figs 2 and 3**. The median frequency of the original VF-ECG (black) is compared to the median frequency of the VF-ECG purged from CPR-artifacts (magenta). In addition, the VF-to-CPR ratio [dB] is shown as a green line (with y-axis tickmarks to the right of the Fig). Here the VF-to-CPR ratio is *estimated *from decomposition of the ECG into VF and CPR by coherent line removal. This procedure also leads to a VF-to-CPR ratio when no CPR is performed, even though rather high (~15 dB). Typical estimated values for VF-to-CPR ratio during CPR are from -15 dB to -5 dB.

Fig 3 shows the windowed Fourier transform of the ECG in the same pig experiment, but now after purging the ECG from CPR-related artifacts using *localized coherent line removal*. The mean and median frequency can now be computed with respect to the extended frequency window [2 Hz, 30 Hz] and follow the "fibrillation band" apart from the last stage of the experiment (> 1100 sec), where no "fibrillation band" is visible any more (probably due to asystole). Fig 4 also shows estimated VF-to-CPR ratios which typically are in a range of -15 dB to -5 dB.

In Fig 4, the median VF-ECG frequency is shown for the original ECG as compared with the median frequency of the VF-ECG purged from CPR-related artifacts by *coherent line removal*. Before start of CPR, these two median frequencies coincide almost perfectly which indicates that *coherent line removal *does not distort VF-ECG when the latter does not contain CPR-related artifacts. After start of CPR, the two median frequencies are rather different and visual inspection shows that the median frequency of the original VF-ECG (containing CPR-related artifacts) is governed by the harmonics of the CPR-artifacts. In the present example, the second harmonic at 5.5 Hz is particularly dominating. The median frequency of the VF-ECG purged from CPR-artifacts by *coherent line removal*, is not governed by the harmonics of CPR-related artifacts, but by the "fibrillation band" which reflects the ventricular fibrillation of the heart of the animal investigated.

**Figure 5:**
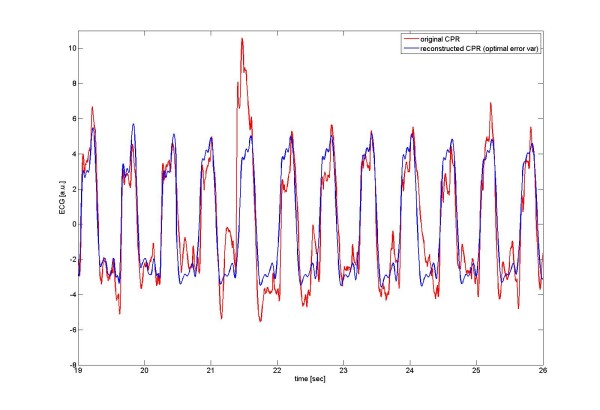
**Illustration of coherent line removal**. Here *human *VF-ECG data (without CPR-related artifacts) have been mixed with ECG containing CPR-artifacts only at a VF-to-CPR ratio of -10 dB, with subsequent purging of CPR-artifacts by *coherent line removal*. The original CPR is shown in red, whereas the CPR-ECG reconstructed by coherent line removal is shown in blue (the criterion being optimal error variance). The improvement in signal-to-noise ratio is 7.3 dB. In this particular example, the human ECG mixed with CPR-ECG had a "fibrillation band" in the frequency range [1 Hz, 5 Hz], which is expected to be difficult to separate from CPR-related artifacts with ~1.8 Hz and harmonics at ~3.7 Hz, ~5.5 Hz etc.

For Fig 5 and Table 1, results from *human out-of-hospital VF-ECGs *are shown, which were mixed with CPR-related ECG artifacts. In Fig 5, the respective VF-to-CPR ratio was chosen to be -10 dB. The reconstructed CPR-ECG is shown for optimal SNR-improvement, i.e., use of optimized parameters for the coherent line removal.

**Table 1:** SNR-gain by coherent line removal

VR-to-CPR ratio [dB]	snr-gain (optimized parameters) n = 168	snr-gain (fixed parameters) n = 168
**-20**	10.3 ± 2.0	9.3 ± 2.4

**-15**	10.5 ± 2.2	9.4 ± 2.4

**-10**	10.5 ± 2.6	9.5 ± 2.5

**-5**	10.2 ± 2.8	9.3 ± 2.5

**0**	8.9 ± 2.9	8.0 ± 2.7

**5**	6.3 ± 3.7	4.9 ± 3.7

**10**	1.7 ± 4.0	-1.0 ± 4.0

For the results of this table, 14 different *human *VF-ECGs (without CPR-related artifacts) have been mixed with 12 different ECG containing CPR-artifacts only at a VF-to-CPR ratio of -20 dB, -15 dB, -10 dB, -5 dB, 0 dB, 5 dB and 10 dB. Typical VF-to-CPR ratios in Fig 4 were in a range of [-20 dB, -5 dB]. In these particular simulations, the human ECG mixed with CPR-ECG had a "fibrillation band" in the frequency range [1 Hz, 5 Hz], which is expected to be difficult to separate from CPR-related artifacts with ~1.8 Hz and harmonics at ~3.7 Hz, ~5.5 Hz etc. After estimation of CPR-artifacts by *coherent line removal*, the SNR-gain can be determined. Since *coherent line removal *can be performed using different parameters (such as the number of harmonics used for the estimation of the CPR-related artifacts in the ECG), one gets snr-gains for every set of parameters. Optimizing the parameters of the algorithm results the SNR-gains shown in column 2 of the Table. Using a the fixed set of default parameters (10 harmonics for analysis and reconstruction and delta = 4) for all different VF-to-CPR ratios still give considerable snr-gain for VF-to-CPR. Results deteriorate if CPR-content in the signal is low (as is the case for a VF-to-CPR ratio of 5 dB). This is what we expected: if almost no CPR-artifacts are present in the signal, the estimation of these very low CPR-artifacts becomes impossible even with most sophisticated techniques.

In this particular example, the human ECG mixed with CPR-ECG had a "fibrillation band" in the frequency range [1 Hz, 5 Hz], which is expected to be difficult to separate from CPR-related artifacts with ~1.8 Hz and harmonics at ~3.7 Hz, ~5.5 Hz etc.

Table 1 refers to 14 different *human out-of-hospital VF-ECG *datasets, mutually mixed with 12 different CPR-ECGs, but now using different choices of VF-to-CPR ratios of -20 dB, -15 dB, -10 dB, -5 dB, 0 dB, 5 dB, and 10 dB. The *optimal *SNR-gains (for optimized parameters of the *coherent line removal *algorithm) were 10.3 ± 2.0 dB, 10.5 ± 2.2 dB, 10.5 ± 2.6 dB, 10.2 ± 2.8 dB, 8.9 ± 2.9 dB, 6.3 ± 3.7 dB, and 1.7 ± 4.0 dB (mean ± std, *n *= 168) in the example used. Optimization by a grid search was done with respect to the parameters which can be chosen for implementation of the *coherent line removal *algorithm (such as the number of harmonics used for the estimation of the CPR-related artifacts in the ECG). If the algorithm is not optimized, but used with the fixed default values of the parameters, the SNR-gains are 9.3 ± 2.4 dB, 9.4 ± 2.4 dB, 9.5 ± 2.5 dB, 9.3 ± 2.5 dB, 8.0 ± 2.7 dB, 4.9 ± 3.7 dB, and -1.0 ± 4.0 dB.

## Discussion

An important point in the development of CPR-artifact reduction algorithms is the use of human VF-ECG data with typical frequencies lying in the same range as the CPR-artifacts themselves. This is not the case in animal experimental data: there often the frequencies of ventricular fibrillation are much higher than the frequencies of CPR-artifacts, simplifying the separation of VF-ECG and CPR-artifacts. Here, we deliberately took human out-of-hospital VF-ECG data with a frequency range of about [1 Hz, 5 Hz], i.e., much lower than in animal data. This frequency range matches well with the frequency range of cardiopulmonary resuscitation of [1.3 Hz, 2 Hz] (= [80/min, 120/min]) and its harmonics. These are compression frequencies near those given in the present resuscitation guidelines (100/min). The animal VF-ECG as used in Figs 1, 2, 3 and 4 served merely the purpose to clearly illustrate the strategy of the coherent line removal method.

The VF-to-CPR ratio (expressed in dB) is negative for very strong CPR-related artifacts and positive, if the CPR-related artifacts are very weak. In case of typical VF-to-CPR ratios which range between -15 dB to -5 dB, the *coherent line removal *algorithm achieves an SNR-improvement of ~9.5.

A typical example would be an original VF-to-CPR ratio of -15 dB, corresponding to a variance ratio *var*(VF):*var*(CPR) = 1:31.6. An improvement by 9.5 dB would result in a restored VF-to-CPR ratio of -5.5 dB, corresponding to a variance ratio *var*(VF):*var*(CPR) = 1:3.5, the variance of the CPR in the signal being reduced by a factor of 8.9.

Another typical example would be an original VF-to-CPR ratio of -10 dB, corresponding to a variance ratio *var*(VF):*var*(CPR) = 1:10. An improvement by 9.5 dB would result in a restored VF-to-CPR ratio of -0.5 dB, corresponding to a variance ratio *var*(VF):*var*(CPR) = 1:1.1, the variance of the CPR in the signal being reduced by a factor of 8.9.

Many studies on CPR artifacts removal used SNR improvement to measure the quality of CPR artifact suppression [19,21,24,35-39]. All these studies were based on the additive data model, i.e., adding a pure artifact signal to the artifact-free ECG, that we adopted. The SNR improvement that we achieved on our testing data is close to that obtained in studies that were based on a two-channel setting, i.e., including additional information such as blood pressure [24,39] and/or compression depth [19,21]. For instance, at the SNR level of -10 dB, we obtained the average SNR improvement of 10.5 dB which is smaller than 12.4 dB and 11.84 dB obtained in refs [19] and [39], respectively, but superior to 10.2 dB obtained in [24]. None of the previous studies has analyzed the SNR improvement for SNR level smaller than -10 dB, i.e., for very large artifacts. It is interesting that in our study the SNR improvement for the SNR levels -15 dB and -20 dB did not increase further and stayed in the same range as the SNR improvement obtained for -5 dB and -10 dB, see Table 1. In contrast, SNR improvement decreases with increasing SNR levels that exceed -5 dB as it was also reported in the previous studies stated above.

Here we used a time window of about 10 sec for application of the *coherent line removal *algorithm (i.e., typically 2 timewindows for a dataset of 20 sec length), which is the reason that single strong CPR-artifacts (with a duration of ~0.6 sec, as shown in Fig 4 in the time window [21 sec, 22 sec]) were not eliminated.

During performance of CPR, automatic analysis of the ECG is difficult, because the predominant part of the signal consists of CPR-artifacts. This is illustrated in Fig 2 by computing the parameters *mean frequency*, the *median frequency *and the *dominant frequency *in the frequency window [4.33 Hz, 30 Hz] [8,10,29,30]. Since this frequency window excludes the CPR-artifacts at ~1.8 Hz and its first harmonic ~3.7 Hz, one might be tempted to consider these parameters (such as the *median frequency*) to be a good indicator of the "fibrillation band" [7,8,11,16,17,40-45]. This is *not *the case. As illustrated in Fig 2, only *before *start of CPR the mean frequency (blue), median frequency (magenta) and dominant frequency (black) in Fig 2 follow very well the "fibrillation band". *After *start of CPR, these parameters are much more governed by the CPR-artifacts than by the "fibrillation band" itself. The *dominant frequency*, in particular, *coincides with the second harmonic of CPR-artifacts *at ~5.5 Hz during the time period [~310 sec, 1410 sec]. During this time period the "fibrillation band" changes dramatically. Nevertheless the dominant frequency is entirely independent of these changes in the "fibrillation band". For the mean frequency and the median frequency, the situation is not so pronounced, but still very unsatisfactory. The *median frequency*, for example, *coincides with the second harmonic of CPR-artifacts *during the time period [~908 sec, ~1410 sec]. We therefore conclude that parameters such as mean, median and dominant frequency are blurred by CPR-artifacts and are therefore not particularly adapted to serve as quantitative indicators for the location of the "fibrillation band".

Fig 3 shows the same pig experiment as Fig 2 again, but now the ECG has been purged from CPR-artifacts by *local coherent line removal*. Again the mean frequency (blue), the median frequency (magenta) and the dominant frequency (black) are shown. The dominant frequency still does not seem to be helpful. The mean and median frequency, on the other hand, now follow closely the "fibrillation band" and can therefore be considered as parameters describing the location of the fibrillation band for the time period [~80 sec, ~1100 sec]. Later than 1100 sec the "fibrillation band" in this pig experiment does not seem to exist any more due to asystole of the heart of the animal. The lower panel of Fig 3 shows that the proportion of the power in the frequency band [0.33 Hz, 2 Hz] as compared to the frequency band [0.33 Hz, 30 Hz] is much lower after filtering of CPR-artifacts than without it. For a large time period [~80 sec, ~1000 sec] this proportion is only about 10% - 20% (instead of 80% - 90%).

Fig 4 shows a comparison (again for the same pig experiment) of the median frequencies before and after CPR-filtering. Observe, in particular, that during the time period [~80 sec, ~310 sec], during which CPR is *not *performed, there is almost a perfect match between the median frequencies computed with/without CPR-filtering. This is to say that the filtering algorithm used (namely *localized coherent line removal*) does not remove anything from the "fibrillation band". During the time period [~80 sec ~1400 sec] where CPR is performed, the median frequency after CPR-filtering (magenta line in Fig 4) follows much better the "fibrillation band" than the median frequency before CPR-filtering (black line in Fig 4).

Optimization of the parameters of the coherent line removal algorithm do *not *give much better results than just using default values (see Table 1). We consider this to be an important result, showing that optimization of the coherent line removal algorithm is not important. Also the default values like *har *and *NumHar *could be changed as long as they are not obviously too small. All values for *har *and *NumHar *larger than our default values would give results rather equal in quality.

When dealing with CPR artefacts of real out-of-hospital cardiac arrests a wide variety of compressions rates are found. Coherent line removal as presented here depends on constant compression rate within the respective chosen time-window.

It is common standard that, when introducing a new method, simple data models (such as additive simulations of CPR artefacts) are used for preliminary testing. Methods need to perform adequately on such simplified data before extending testing on a heterogenous dataset. Most of the authors hitherto have used additive models of VF. Also, to the best of our knowledge, the SNR is the basic criterion for classifying the quality of a CPR-removal algorithm. In our recently published paper [46], we investigated the efficiency of various two-channel methods for CPR-artefact removal in *non*-shockable rhythms: for such ECGs, two-channel methods could not reduce CPR artefacts without affecting the rhythm analysis for shock recommendation. For the coherent line removal algorithm the same is true, i.e., the efficiency and quality of CPR-artefact removal for *non*-shockable rhythms is not satisfactory. Possibly a better understanding of the spectral distribution of rhythms other than VF is needed for future adaption of the coherent line algorithm.

From a computational point of view, the coherent line removal algorithm is very fast. It could probably be implemented on a Coldfire processor containing a floating point unit. Such Coldfire processors are commercially available, but only when ordering large amounts (> 10000 units). The coherent line removal algorithm is, in particular, much faster than the Kalman filter algorithm presented in ref [24].

## Conclusions

The *localized coherent line removal *algorithm reduces CPR-artifacts in VF-ECG, but does not eliminate them. It uses the ECG-channel only, without any additional information (like blood pressure). Our SNR-improvements are in the same range as offered by multichannel methods of Rheinberger et al. [24], Husoy et al. [20] and Aase et al. [21]. In refs. [20,21] the authors dealt with different rhythms (VF and VT) whereas we dealt with VF, exclusively. Additional developments are necessary before the algorithm can be tested in real CPR situations.

## Appendix: Short description of the coherent line removal algorithm

In Ref [33], the *coherent part y*(*t*) of a signal *s*(*t*) = *y*(*t*) + *n*(*t*) is described as

where the overbar denotes complex conjugation and where *α*_*k *_are appropriate coefficients. Here *m*(*t*) is a nearly monochromatic signal,

with slowly varying amplitude *r*(*t*) and frequency *f*_0_(*t*), and with . The number *M *of considered harmonics is one of the parameters of the coherent line removal algorithm.

In the present application, the *coherent part y*(*t*) of the ECG-signal is identified with its CPR-artifacts, corresponding to the function estimate_CPR_(*t*) in the Methods Section. The frequency *f*_0_(*t*) is the frequency of resuscitation (e.g., 110/min = 1.83 Hz).

We use a *localized *version of the approach from Ref [33], using timewindows of 10.24 sec length. *Localization *is necessary, because CPR-related artifacts change in amplitude and frequency, and may disappear at all when no resuscitation is performed. *Localization *means that the coefficients *α*_*k *_depend on the specific timewindow, e.g., [0 sec, 10.24 sec], [10.24 sec, 20.48 sec], [20.48 sec, 30.72 sec] etc. If no resuscitation artifacts are present in a timewindow, then we have *α*_*k *_= 0 for all harmonics *k *= 1, 2, ..., *M*.

The *step-in-frequency *is given by

The *width of the frequency window *used for the estimation of the parameters *α*_*k *_is related to the parameter *δ *by

For our default value *δ *= 4, the *width of the frequency window *is therefore given as

In the frequency space, the decomposition *s*(*t*) = *y*(*t*) + *n*(*t*) is given as

where the hat *ŝ *= *ŝ*(*v*) indicates the Fourier transform (depending on frequency *v*) of a function *s *= *s*(*t*). Here *n *= *n*(*t*) corresponds to the proper VF-ECG without the CPR-related artifacts. Restricting oneself to the frequency window around the *k*^th^-harmonic, one gets

Applying the inverse Fourier transform, this leads to

Using the abbreviations

and

one gets

Considering the VF-ECG *n*(*t*) and the respective restrictions *n*_*k*_(*t*) to a frequency window around the *k*^th^-harmonic as a stochastic processes with ensemble mean value zero, ⟨*n*_*k*_(*t*)⟩ = 0, leading to

Hence multiplication of the stochastic processes *B*_*k*_(*t*) by appropriate scalars Γ_*k *_leads to stochastic processes

which all have the *same *ensemble mean value

The values of the scalars Γ_*k *_can be obtained from a least square method, comparing the first harmonic with the other harmonics considered, and taking Γ_*k *_as the scalar which leads to the minimal value of the ensemble expectation . This is achieved by setting

From the values of Γ_*k *_one may compute the values for the *α*_*k*_, namely *α*_*k *_= (*α*_1_Γ_*k*_)^*k*^.

Finally the interference *m*(*t*) is *reconstructed *as a linear combination

such that it has the same mean and minimum variance. Here . Note that the number *M *of harmonics for analysis may be different from the number *N *of harmonics for reconstruction. The parameters *M *and *N *may be used for optimization of the *coherent line removal *algorithm. This leads to

which allows to estimate the coherent part of the original signal.

## Competing interests

The authors declare that they have no competing interests.

## Authors' contributions

The human study was planned and performed by MB and AA. The animal experiments were planned and performed by WL. The MATLAB mfile for coherent line removal was written by AK, with later modifications by AA. The compilation of human ECG-data (from different traces recorded on the MRL-defibrillator) was performed by TN, who also prepared a respective database and programmed the MATLAB toolbox for scoring and handling of the data. The toolbox was subsequently modified by AKu. The choice of the data and the respective MATLAB-experiments were performed by AA with support by AK, TW and MG. The distributed computing of the coherent line algorithm was implemented by AKu. The manuscript was written by AA, TW, MB and WL. All authors approved the final version of the manuscript.
